# MicroRNA-198 inhibits proliferation and induces apoptosis by directly suppressing FGFR1 in gastric cancer

**DOI:** 10.1042/BSR20181258

**Published:** 2019-06-10

**Authors:** Junxia Gu, Xiaozhen Li, Hui Li, Zhe Jin, Jianjun Jin

**Affiliations:** 1 Department of Digestive, The First Affiliated Hospital, and College of Clinical Medicine of Henan University of Science and Technology, Luoyang City, Henan Province, 471003, P.R. China; 2 Endoscopy Center, The First Affiliated Hospital, and College of Clinical Medicine of Henan University of Science and Technology, Luoyang City, Henan Province, 471003, P.R. China

**Keywords:** FGFR1, gastric cancer, gene therapy, miR-198, miRNA

## Abstract

MicroRNAs (miRNAs) are increasingly recognized as important therapeutic targets in cancer. Here we aim to investigate the role of miR-198, a broad-spectrum tumor suppressor, in gastric cancer (GC). MiR-198 overexpression was achieved by transfection of miR-198 mimics, followed by evaluation of cell viability using cell-counting kit 8. Cell cycle arrest and apoptosis were assessed by Annexin-V-FITC/Propidium Iodide (PI) staining flow cytometry respectively. The target of miR-198 was identified by bioinformatical analysis and confirmed by dual-luciferase assay, along with real-time PCR and Western blot analyses of target gene expression after transfection of miR-198 mimics. GC tissues were characterized by miR-198 down-regulation. Restoration of miR-198 expression attenuated GC cell proliferation and colony formation, meanwhile inducing significant G_0_/G_1_ arrest. Furthermore, combinatory therapy of cisplatin and miR-198 induced greater anti-tumor effects than treatment with cisplatin single therapy. We also identified fibroblast growth factor receptor 1 (FGFR1) as a direct target gene of miR-198. Furthermore, FGFR1 silencing elicited a similar tumor-suppressive effect as miR-198 overexpression. FGFR1 overexpression antagonized the anti-tumor effects of miR-198 overexpression. MiR-198/FGFR1 axis plays an important role in proliferation and apoptosis of GC. Therapies targeted to miR-198 can potentially improve GC treatment.

## Introduction

Gastric cancer (GC) is a prevalent cancer and a leading cause of cancer-related deaths around the globe, particularly in developing countries [1]. Despite decreasing incidence rate over the years and recent advances in GC diagnostic technologies, a large population of GC patients are diagnosed at an advanced stage due to the lack of initial symptoms [2]. Surgery, chemotherapy and radiotherapy are main treatment strategies for GC. While the survival rates of patients with locoregional cancers are generally high, the overall survival rate of GC patients is still poor (5-year survival rate = 31%) [1], due to late diagnosis of the disease and the inherent or acquired resistance of GC, leading to suboptimal therapeutic response [3]. Targeted therapies are commonly used for GC treatment, and to overcome cancer resistance, researchers are in seek of new druggable targets to improve the therapeutic outcome [4].

A class of new cancer targets is microRNAs (miRNAs), which are non-coding small, single-stranded RNAs that regulate a broad-spectrum of genes involved in developmental and oncogenic pathways. Their regulatory role is mediated through target mRNA degradation of translation suppression after binding with the 3′-untranslated regions (3′-UTRs) [5]. Abnormal expression of miRNAs was found to be correlated with cancer and many diseases [6–8]. This has fueled the development of miRNAs as biomarkers for GC diagnosis and inspired novel therapeutic strategies that involve the delivering molecules that suppress or promote the expression of these cancer-specific miRNAs [7–9]. miR-198 is one such therapeutic miRNAs with potent therapeutic efficacy in breast cancer, hepatocellular carcinoma, colorectal cancer, lung cancer etc [10–14]. A recent study has reported the down-regulation of miR-198 as a biomarker of GC [9]. However, the mechanism of miR-198 regulation in cancer is not fully elucidated and it remains unclear whether therapies based on miR-198 can exert therapeutic effects in GC.

Herein, the purpose of the present study is to characterize miR-198 as a therapeutic molecule in GC and to elucidate the underlying molecular mechanism. Expression of miR-198 in GC and normal tissue was investigated to verify miR-198 as a diagnostic biomarker in GC. miR-198 mimic was transfected in GC SGC-7901 cells to evaluate its effects on cancer proliferation, cell cycle arrest and apoptosis. The role of a critical oncogene fibroblast growth factor receptor 1 (FGFR1), which was reported as a target of miR-198 in lung cancer [15], was also investigated. The results in our study could provide a novel tool for GC diagnosis and therapy and potentially improve the clinical management of this lethal disease.

## Materials and methods

### Tissue sample collection and cell culture

Primary GC tissue samples and adjacent non-tumor gastric tissues of 118 cases were collected from The First Affiliated Hospital of Henan University of Science and Technology. The present study was conducted in compliance to the recommendations of the World Medical Association Declaration of Helsinki and approved by the Institutional Review Board of The First Affiliated Hospital of Henan University of Science and Technology. Written informed consent was acquired from each participant. Tissue samples were snap-frozen in liquid nitrogen after surgical removal and stored at −80°C. The GC cell line, SGC-7901 and normal human gastric epithelial cells (NGEC) were acquired from the Institute of Biochemistry and Cell Biology, Chinese Academy of Sciences (Shanghai, China). The cells were maintained in DMEM, supplemented with 10% fetal bovine serum (FBS; HyClone, U.S.A.), 100 U/ml penicillin and 100 μg/ml streptomycin. The cells were incubated in 5% CO_2_ at 37°C.

### Synthesis and transfection of miRNA/siRNA and plasmid DNA

miR-198 mimics, siRNA targeting human FGFR1 mRNA and negative controls were designed and synthesized by Shanghai GenePharma Company (Shanghai, China). Cells were seeded into 96-well plates in antibiotic-free growth medium at the density of 4 × 10^3^ cells per well. Transfection with human miR-198 mimics or negative control (control) was performed when the cells reached 70–80% confluence. For FGFR1 or scrambled siRNA, cells transfection was mediated by Lipofectamine 2000 (Invitrogen, U.S.A.) when cells reached 30–40% confluence, in accordance with the manufacturer’s protocol. The pCDNA3.1-FGFR1 plasmid, which encompassed the entire FGFR1 coding sequence but lacked the normal 3′-untranslated sequence, was purchased from Shanghai GenePharma Company (Shanghai, China). Experiments were performed 72 h after transfection.

### miRNA detection

Total RNA was extracted using TRIzol (Invitrogen). Mature miRNAs were reverse transcribed into cDNA and qRT-PCR was performed on a thermocycler using the TaqMan microRNA Assays Kit (Applied Biosystems) according to the manufacturer’s manual. The data were normalized to U6 expression. Data are reported based on three independent replicates.

### Cell proliferation assay

Cell proliferation was determined using the Cell Counting Kit-8 (CCK-8) assay kit (Takara, Dalian, China). Cells were seeded at a density of 3 × 10^4^ per well in 96-well plates, and each well was transfected with miR-198 mimic (50 nM), inhibitor (100 nM) or the proper negative control. At 72 h after transfection, the absorbance at 450 nm was read using an iMark Microplate Absorbance Reader (Bio-Rad, Hercules, CA, U.S.A.).

### Cell cycle assay

At 72 h after transfection, cells were collected and washed twice at 4°C with PBS supplemented with 0.5% BSA. Cells were harvested by centrifugation and re-suspended in 300 μl PBS, followed by fixation with 700 μl of 100% ethanol at 4°C for 24 h. To remove ethanol, cells were washed twice in ice-cold PBS and then resuspended in a propidium iodide (PI) solution containing 50 μg/ml RNase and 100 μg/ml PI and (Sigma, U.S.A.) at 37°C for 30 min in dark. Cell number in each cell cycle was measured using Calibur Flow Cytometers and Cell Quest software.

### Analysis of clonogenicity *in vitro*

Aliquots of viable SGC-7901 cells (1000 per well) transfected with miR-198 mimic or miR-NC were plated in six-well plates at 24 h after transfection and cultured in growth medium for 2 weeks. The number of colonies, defined by cell clusters that contained more than 50 cells, were stained with Crystal Violet and manually counted under a light microscope.

### Apoptosis analysis by Annexin-V FITC/PI double staining

The cells were treated with 5 μM cisplatin. To identify apoptotic cells, Annexin V and PI staining was performed using an Annexin V-FITC Apoptosis Detection kit (Becton, Dickinson and Company, San Jose, CA, U.S.A.). After treatment, 5 × 10^5^ cells were pelleted by centrifugation at 1500 rpm for 5 min. The cells were then re-suspended in 200 μl of binding buffer and incubated with 5 μl FITC Annexin V and 1 μl PI solution for 30 min at RT. Apoptosis was detected using a Calibur Flow Cytometer (Becton). Apoptotic cells were defined by positive staining of Annexin V and negative staining of PI.

### TUNEL assay

TUNEL assay with the *In Situ* Cell Death Detection Kit (TUNEL fluorescence FITC kit, Roche, Indianapolis, IN, U.S.A.) was used to evaluate DNA fragmentation of individual cells. Cells cultured on coverslips were washed with PBS containing 137 mM NaCl, 2.7 mM KCl, 4.3 mM Na_2_HPO_4_ and 1.4 mM KH_2_PO_4_, pH 7.4, followed by fixation in 4% paraformaldehyde solution (PFA) for 1 h at 4°C. Cells were then permeabilized using 0.1% Triton X-100 solution for 2 min, and cells were incubated in freshly prepared TUNEL reaction mixture for 1 h at 37°C in dark. The coverslips were then washed with PBS. Following this, the coverslips were mounted on slides with Prolong anti-fade solution (Invitrogen, U.S.A.) and TUNEL staining was analyzed with a fluorescence microscopy (Eclipse 80i; Nikon Co., Tokyo, Japan).

### Luciferase reporter assay

The 3′-UTR of FGFR1 mRNA, which contained the predicted miR-198 binding site based on bioinformatical analysis using TargetScan, starBase and miRmap databases, was amplified by PCR using the Takara PCR Amplification Kit (Takara, Dalian, China). The Quick Mutagenesis Stratagene kit (Stratagene, La Jolla, CA) was used to create mutant 3′-UTR of EGFR1 mRNA. The PCR products were then enzymatically digested and cloned into the psiCHECK-2 reporter vector (Promega, U.S.A.). Cells (0.5 × 10^5^) were seeded in 24-well plates and cultured for 24 h. Reporter plasmids (200 ng psiCHECK-2-FGFR1-wild or psiCHECK-2-FGFR1-mut) and 100 nmol/l miR-198 mimics were co-transfected into SGC7901 cells mediated by Lipofectamine 2000 (Invitrogen). After 48 h, the cells were lysed and reporter activity was determined using a Dual Luciferase Reporter Assay Kit (Promega, Madison, WI, U.S.A.), according to the manufacturer’s instructions.

### Western blotting

Western blotting was performed using standard methods. Membranes were probed with polyclonal rabbit antibodies against anti-FGFR1 (1:500; Abcam, Cambridge, MA, U.S.A.). The proteins on membranes were then stripped and re-probed with an anti-GAPDH rabbit polyclonal antibody (Abcam) as a loading control. The blot was developed using enhanced chemiluminescence solution (Beyotime, Haimen, China) and photographed using the FluorChem imaging system (Alpha Innotech Corp., San Leandro, CA, U.S.A.). The intensity of each spot was analyzed with AlphaEaseFC software.

### Statistical analysis

All data are represented as the mean ± standard deviation of at least three independent experiments. The results were analyzed using a two-tailed Student’s *t* test and chi-square test was used for the analysis of correlation between miR-198/FGFR1 expression and different clinicopathological features in GC patients. Results were considered statistically significant at *P*<0.05.

## Results

### MiR-198 is down-regulated in human GC tissues and cell lines

**Figure 1:**
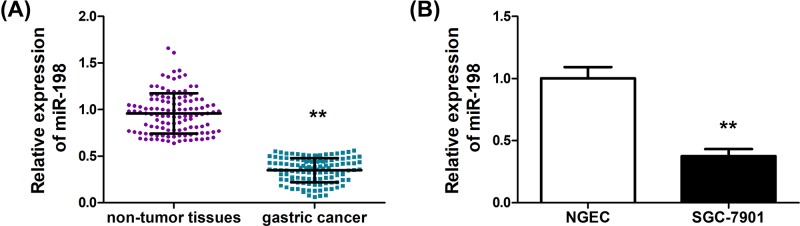
MiR-198 is down-regulated in human GC tissues and cell lines (**A**) MiR-198 expression in primary GC tissues compared with paired adjacent normal tissues from 118 patients. (**B**) Real-time PCR analysis of miR-198 expression in NGEC and GC SGC-7901 cell lines. Bars denote the mean of three independent experiments. ^**^P<0.01.

To characterize miR-198 as a biomarker in human GC tissues, qRT-PCR was first performed and the expression levels of miR-198 in GC tissues and adjacent normal tissues were compared. As shown in Figure 1A, a marked down-regulation of miR-198 in GC tissues can be observed (*P*<0.01). Further, analysis of miR-198 expression in normal gastric cell NGEC and GC cell SGC-7901 was conducted. Consistently, miR-198 was significantly lower in SGC-7901 than that in NGEC (*P*<0.01). In other GC cell lines, including AGS, MGC803, MKN-28 and BGC823, a down-regulation of miR-198 was observed (Supplementary Figure S1A). These data confirmed that down-regulation of miR-198 is correlated with GC tumorigenesis (Figure 1B). The level of miR-198 and various clinicopathological characteristics of GC are summarized in Supplementary Table S1. The expression of miR-198 in GC patients did not correlate with age, gender or cancer differentiation. However, statistically significant correlations were found between low levels of miR-198 with tumor size (*P*=0.025), tumor depth (*P*=0.016), lymph node metastasis (*P*<0.01) and clinical stage (*P*<0.01). Moreover, high levels of FGFR1 were correlated with tumor size (*P*<0.01), tumor depth (*P*<0.01), lymph node metastasis (*P*=0.001) and clinical stage (*P*<0.01) (Supplementary Table S2).

### MiR-198 suppresses growth and promotes apoptosis in SGC-7901 cells

**Figure 2:**
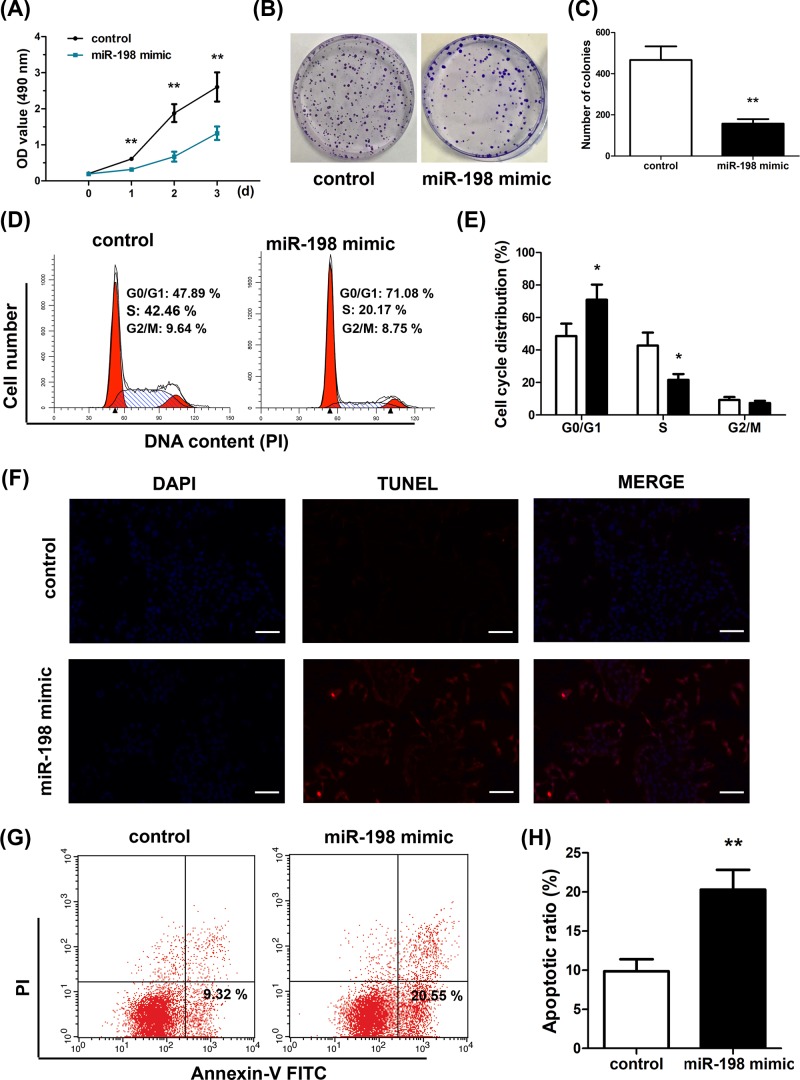
MiR-198 suppresses growth and promotes apoptosis in SGC-7901 cells (**A**) Cell proliferation was measured using CCK-8 assays. Overexpression of miR-198 inhibited SGC-7901 cell proliferation. (**B**,**C**) Colony formation ability was determined using colony formation assays. Overexpression of miR-198 inhibited the colony formation ability of SGC-7901 cells. (**D**,**E**) Cell-cycle distribution was detected using flow cytometry. (**F**) Apoptosis was detected using TUNEL staining assays. (**G**,**H**) Apoptosis was detected by Annexin-V FITC/PI double-staining, apoptotic cells were defined as Annexin V-positive/PI-negative, overexpression of miR-198 enhanced apoptosis induced by 5 μM cisplatin in SGC-7901 cells. ^*^P<0.05; ^**^P<0.01.

To correlate phenotypic change in GC cells with miR-198 expression, we overexpressed miR-198 in SGC-7901 cells through miR-198 mimic transfection. Following this, proliferation, colony formation, cell cycle arrest and apoptosis were monitored. CCK-8 assay indicated that after miR-198 mimic transfection, the proliferation of SGC-7901 cells was prominently suppressed (*P*<0.01) (Figure 2A). Colony formation assay, as shown in Figure 2B,C, showed that SGC-7901 cells with miR-198 overexpression had fewer colonies formed (*P*<0.01). Similar findings were observed for AGS, MGC803, MKN-28 and BGC823 cells with miR-198 overexpression (Supplementary Figure S1B). miR-198 overexpression also resulted in higher cell cycle arrest, evidenced by larger number of cells at G_0_/G_1_ stage (Figure 2D,E) based on PI staining of the cells followed by flow cytometry analysis. TUNEL staining and Annexin-V-FITC/PI staining flow cytometry also demonstrated that cells with miR-198 overexpression exhibited higher levels of cell apoptosis under cisplatin treatment (Figure 2F–H), corroborating the role of miR-198 as a gastric tumor suppressor.

### FGFR1 is a direct target of miR-198 in GC cells

**Figure 3:**
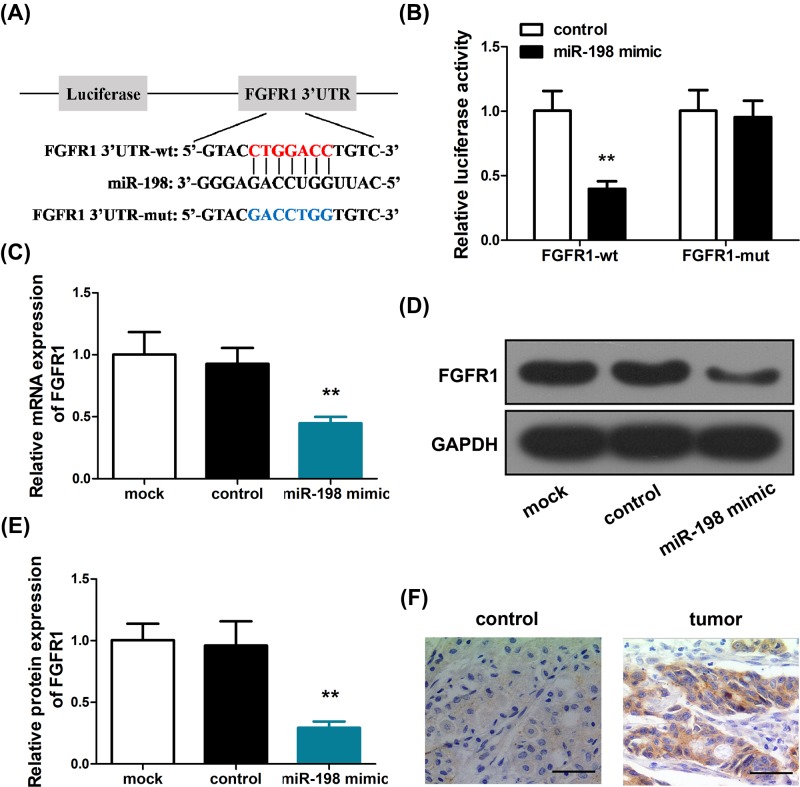
FGFR1 is a direct target of miR-198 in GC cells (**A**) TargetScan predicted a miR-198 target sequence in the 3′-UTR of FGFR1 mRNA. (**B**) FGFR1-3′UTR-wild or FGFR1-3′-UTR-mut plasmids were transfected into miR-198-treated SGC-7901 cells and assayed for luciferase activity. (**C**) The mRNA levels of FGFR1 were detected by RT-PCR in SGC-7901 cells transfected with miR-198 mimics. (**D**,**E**) FGFR1 protein levels were detected by Western blotting in SGC-7901 cells transfected with miR-198 mimics. (**F**) FGFR1 expression was detected by immunohistochemistry in GC tissue and non-tumor gastric tissues. ^**^P<0.01.

To clarify the mechanism of miR-198 regulation in GC, we performed TargetScan analysis to search for targets of miR-198 and found that the 3′-UTR of FGFR1 interacts with miR-198 (Figure 3A). This finding was confirmed by bioinformatical analysis using another two databases, starBase and miRmap. Hence, we hypothesized that miR-198 exerts its tumor-suppressive effect in part through the regulation of FGFR1, which is a putative oncogene. To test this hypothesis, we designed a mutant sequence of the FGFR1 3′-UTR (Figure 3A), and utilized dual-luciferase assay to probe the interaction between miR-198 and the wild-type/mutant 3′-UTR of FGFR1. As shown in Figure 3B, miR-198 mimic transfection dramatically decreased the luciferase activity of wild-type FGFR1 compared with control, whereas no significant changes of luciferase activity were observed in the miR-198 mimic transfected cells with mutant FGFR1. Quantitative RT-PCR (Figure 3C) and Western blot (Figure 3D,E) indicated that transfection of miR-198 mimic also induced a down-regulation of FGFR1 in SGC-7901 cells relative to control, while transfection of miR-NC did not induce significant changes of FGFR1 expression. Immunohistochemical analysis of tumor tissues indicated an up-regulation of FGFR1 in tumor tissues (Figure 3F). Collectively, these evidences confirmed that miR-198 suppresses FGFR1 expression as a mechanism for GC regulation.

### MiR-198 mimics and siFGFR1 elicit similar repressive effects

**Figure 4:**
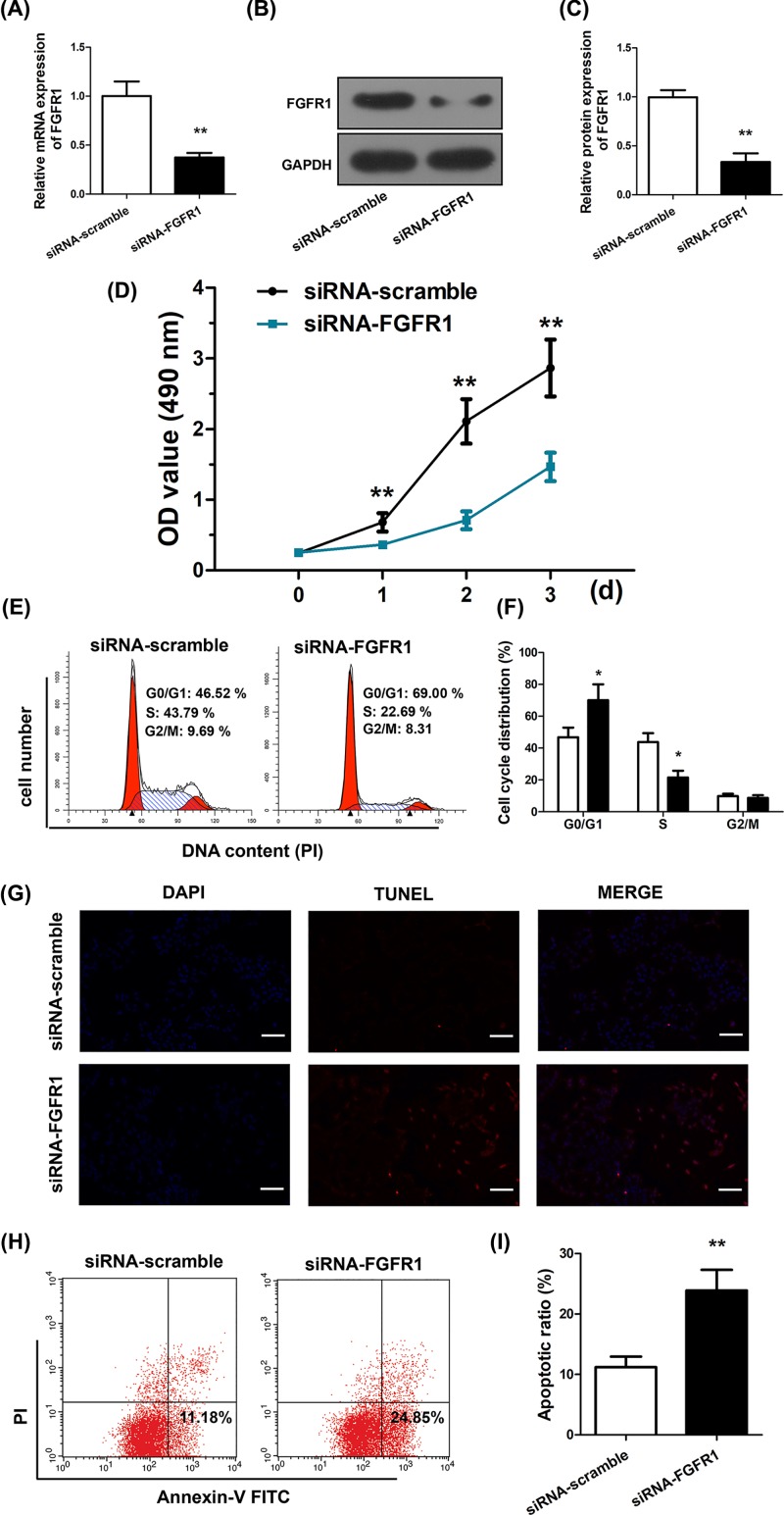
MiR-198 mimics and siFGFR1 elicit similar repressive effects (**A**) FGFR1 mRNA levels were detected by RT-PCR in SGC-7901 cells transfected with siFGFR1 or scrambled siRNA. (**B**,**C**) FGFR1 protein levels were detected by Western blotting in SGC-7901 cells transfected with siFGFR1 or scrambled siRNA. (**D**) Cell proliferation was measured using CCK-8 assays in SGC-7901 cells transfected with siFGFR1 or scrambled siRNA. (**E**,**F**) Cell-cycle distribution was detected by flow cytometry in SGC-7901 cells transfected with siFGFR1 or scrambled siRNA. (**G**) Apoptosis was detected using TUNEL staining assays in SGC-7901 cells transfected with siFGFR1 or scrambled siRNA. (**H**,**I**) Apoptosis was detected using Annexin V FITC/PI double-staining in SGC-7901 cells transfected with siFGFR1 or scrambled siRNA, apoptotic cells were defined as Annexin V-positive/propidium-negative. ^*^P<0.05; ^**^P<0.01.

Given that FGFR1 is a target of miR-198, we next examined if FGFR1 silencing could result in similar GC suppression as miR-198. To this end, we transfected an FGFR1 siRNA (Supplementary Figure S1C), siFGFR1, which resulted in reduction in FGFR1 mRNA (Figure 4A) and protein (Figure 4B,C) levels. Consequently, SGC-7901 cells with siFGFR1 transfection led to suppressed proliferation as demonstrated in CCK-8 assay (Figure 4D). Concurrently, siFGFR1 also promoted cell cycle arrest (Figure 4E,F), as revealed by PI staining and flow cytometry. TUNEL staining (Figure 4G) and Annexin-V-FITC/PI staining flow cytometry (Figure 4H,I) also suggested increased cell apoptosis under FGFR1 silencing. Therefore, siFGFR1 is similar to miR-198 mimics in suppressing GC cells.

### Overexpression of FGFR1 reverses the repressive effects caused by miR-198 mimics

**Figure 5:**
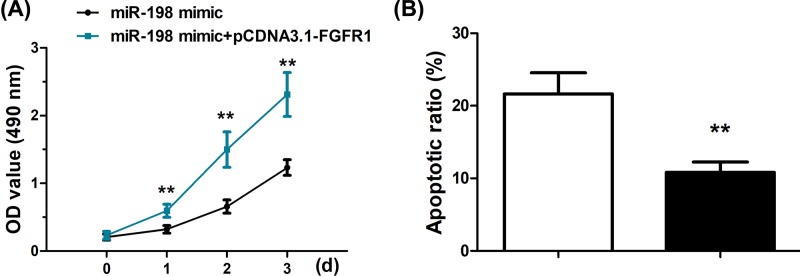
Overexpression of FGFR1 reverses the repressive effects caused by miR-198 mimics (**A**) Cell proliferation was measured using CCK-8 assays in SGC-7901 cells transfected with miR-198 mimics alone or co-transfected with miR-198 mimics and pCDNA3.1-FGFR1. (**B**) Apoptosis was detected by Annexin V FITC/PI double-staining in SGC-7901 cells transfected with miR-198 mimics alone or co-transfected with miR-198 mimics and pCDNA3.1-FGFR1. ^**^P<0.01.

To gain further insight into the interaction between miR-198 and FGFR1, we next examined if FGFR1 overexpression, mediated by transfection of pCDNA3.1-FGFR1, could abrogate the cancer-suppressive effects of miR-198. FGFR1 overexpression was confirmed both at mRNA and protein level (Supplementary Figure S2). Proliferation assay was conducted in cells transfected with both miR-198 mimics and pCDNA3.1-FGFR1, which demonstrated significantly higher proliferation rate that those solely with miR-198 mimic transfection (Figure 5A). Apoptosis rate of the cells with both miR-198 mimics and pCDNA3.1-FGFR1 transfection was also lower (Figure 5B). These corroborated that FGFR1 overexpression antagonizes the tumor-suppressive effect of miR-198 and FGFR1 is a target of miR-198.

## Discussions

Accumulating evidences suggested that miR-198 down-regulation is associated with the pathogenesis of many human diseases and cancers. Our results confirmed that miR-198 down-regulation is correlated with GC. Current diagnosis of GC is mostly based on imaging, endoscopy or biopsy methods in symptotic patients [16,17]. However, factors, such as invasiveness, accessibility and cost-effectiveness limit the development of these methods for GC screening [18], which is critical for early detection of GC and reduction in mortality rates. Comparatively, diagnostic strategies based on levels of serum RNAs, such as long non-coding RNAs (lncRNAs) and miRNAs, have been increasingly studied in recent years, potentiating the use of a simple blood test for cancer screening [18,19]. Our study adds an important tool for GC diagnosis based on evaluating miR-198 expression. Further study that characterizes the correlation of serum miR-198 levels and GC is warranted to demonstrate the practicality of this approach for GC screening.

The down-regulation of miR-198 in GC tissues also pointed to the hypothesis that miR-198 down-regulation is essential for the aggressive phenotypes of GC. We therefore next examined whether restoration of miR-198 expression, through miR-198 mimic transfection, could attenuate the GC progression. Indeed, our data echoed previous reports on the anti-tumor effects of miR-198 mimic transfection in other cancers [10,18] that delivery of miR-198 in GC cells exerted potent tumor suppression. To date, no reports have described therapeutic effects of miR-198 in GC, and our study represents the first report on the effects of miR-198 in GC cell proliferation, cell cycle arrest and apoptosis. Given the advances on gene delivery technologies [20], cancer therapy based on miR-198 mimics delivery could be used to precisely suppress oncogenes regulated by miR-198. But one limitation of our study is that *in vivo* transfection of miR-198 mimics in GC has not been performed. Based on our *in vitro* evidence on the tumor-suppressive function of miR-198 in GC, it is imperative to validate the potential of miR-198 mimics in GC treatment *in vivo* to facilitate translation of this gene therapy approach into clinics.

As a mechanistic study, we also conducted bioinformatical analysis to identify the target of miR-198 and validated FGFR1 as the effector of miR-198 regulation. Using dual-luciferase assay, we confirmed that miR-198 suppressed FGFR1 levels by binding to the 3′-UTR region of the oncogene. Previous studies have indicated that other miRNAs, such as miR-133b [21], which is another tumor suppressor, also targets FGFR1 to suppress GC. Being a member of the fibroblast growth receptor family, FGFR1 acts as a receptor tyrosine kinase, which interacts with fibroblast growth factors to trigger a cascade of downstream signaling pathways that govern cell proliferation, survival, migration and differentiation. Activation of FGFR1 has been shown linked with a large body of human cancers [22]. Additionally, epithelial–mesenchymal transition (EMT), an important process for cancer dissemination and metastasis, is also induced by FGFR1 activation [23]. These evidences prompted the use of FGFR1 as a therapeutic target for the treatment of cancers. For example, FGFR kinase inhibitors such as dovitinib, brivanib, SU‐6668 and BIBF112 have already been shown to inhibit tumor proliferation and are now undergoing clinical testing in cancer therapy [24]. The FGFR1 silencing mediated by siRNAs, as demonstrated in our study, is also one of the approaches to deactivate FGFR1 in cancer. Our data indicated that miR-198 is also an inhibitor of FGFR1 similar to the aforementioned drugs. However, it should also be noted that miR-198 regulate a broad-spectrum of biological processes and the targets of miR-198 are not limited to oncogenes. MiR-198 overexpression has been found in diseases such as preeclampsia and anencephaly [13]. Moreover, FGFR1 is involved in important processes such as mitogenesis, cell maturation, blood vessel formation, wound healing, embryonic and nervous system development [15], and the side effects of miR-198 gene therapy should also be scrutinized.

## Conclusion

In sum, we herein verified miR-198 down-regulation as a biomarker of GC. Transfection of miR-198 mimics was shown to prominently attenuate GC cell proliferation, meanwhile inducing cell cycle arrest and apoptosis. TargetScan study identified FGFR1 as a target of miR-198, and the interaction of miR-198 with FGFR1 was verified using qRT-PCR, Western blot and immunohistochemistry. These evidences suggested that miR-198 is a gene with important diagnostic and therapeutic potential in GC.

## Supporting information

**Supplementary Figure S1:** 

**Supplementary Figure S2:** 

**Supplemental Table S1:** Correlation between miR-198 expression and different clinicopathological features in gastric cancer patients

**Supplemental Table S2:** Correlation between FGFR1 expression and different clinicopathological features in gastric cancer patients

## Abbreviations

CCK-8: cell counting kit-8
FGFR1: fibroblast growth factor receptor 1
GC: gastric cancer
miRNA: microRNA
NGEC: normal human gastric epithelial cell
PI: propidium iodide
3′-UTR: 3′-untranslated region
