# Supplementary material for: MicroRNA-198 inhibits proliferation and induces apoptosis by directly suppressing FGFR1 in gastric cancer
Source: Biosci Rep. 2019 Jun 10;39(6):BSR20181258. doi: 10.1042/BSR20181258 (PMC6558723; doi:10.1042/BSR20181258)
Supplement: Supplementary file 1 [file bsr-2018-1258_suppS1.pdf]

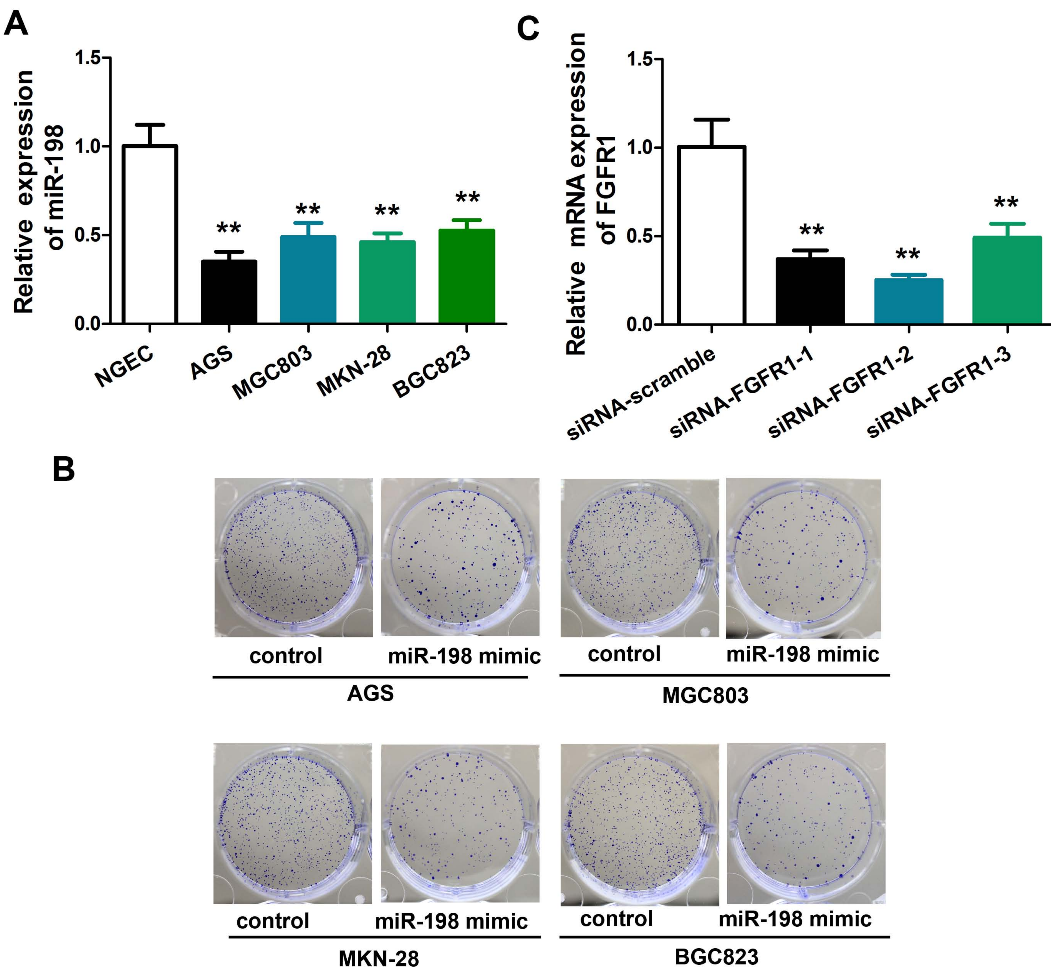
A
Relative expression of miR-198
1.5
1.0
0.5
0.0
**
**
**
**
NGEC
AGS
MGC803
MKN-28
BGC823
C
Relative mRNA expression of FGFR1
1.5
1.0
0.5
0.0
**
**
**
siRNA-scramble
siRNA-FGFR1-1
siRNA-FGFR1-2
siRNA-FGFR1-3
B
control
miR-198 mimic
AGS
control
miR-198 mimic
MGC803
control
miR-198 mimic
MKN-28
control
miR-198 mimic
BGC823

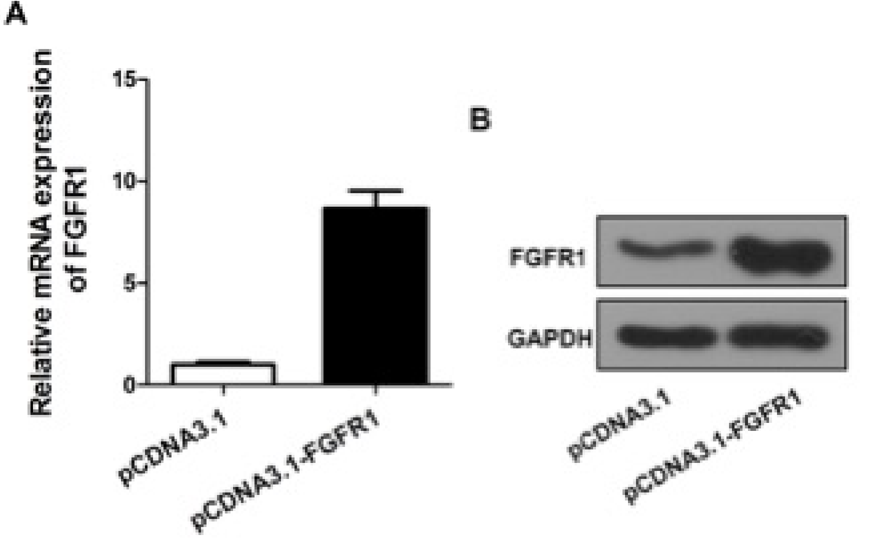
A
Relative mRNA expression of FGFR1
15
10
5
0
pCDNA3.1
pCDNA3.1-FGFR1
B
FGFR1
GAPDH
pCDNA3.1
pCDNA3.1-FGFR1

Supplementary Table 1. Correlation between miR-198 expression and different clinicopathological features in gastric cancer patients

	**Low miR-198 expression**	**High miR-198 expression**	***p* value**
Age			
≥60	38	41	0.696
<60	21	18	
Gender			
Male	31	35	0.578
Female	28	24	
Differentiation			
Well-moderate	18	24	0.337
Poor	41	35	
Tumor size			
≥5 cm	41	28	0.025
<5 cm	18	31	
Invasion depth			
T1, T2	19	33	0.016
T3, T4	40	26	
TNM stage			
I/II	15	30	＜0.01
III	45	28	
Lymphatic metastasis			
Negative	12	28	＜0.01
Positive	46	32	

Supplementary Table 2. Correlation between FGFR1 expression and different clinicopathological features in gastric cancer patients

	**Low FGFR1 expression**	**High FGFR1 expression**	***p* value**
Age			
≥60	39	40	0.564
<60	17	22	
Gender			
Male	32	34	0.578
Female	22	30	
Differentiation			
Well-moderate	20	22	0.848
Poor	34	42	
Tumor size			
≥5 cm	24	45	＜0.01
<5 cm	35	14	
Invasion depth			
T1, T2	34	18	＜0.01
T3, T4	25	41	
TNM stage			
I/II	31	14	＜0.01
III	27	46	
Lymphatic metastasis			
Negative	28	12	＜0.01
Positive	30	48	
